# Herba Epimedii and Increased Opioid Cravings While on Buprenorphine: A Case Report

**DOI:** 10.7759/cureus.51886

**Published:** 2024-01-08

**Authors:** Heather Burke, Bernard A Sarmiento, Matthew Gunther, Richard Czuma, Cory Klippel, Shixie Jiang

**Affiliations:** 1 Department of Psychiatry and Human Behavior, Brown University, Providence, USA; 2 College of Medicine, University of Central Florida, Orlando, USA; 3 Department of Psychiatry, Stanford University School of Medicine, Palo Alto, USA; 4 Department of Psychiatry, Edward Hines, Jr. Veterans Administration Hospital, Hines, USA; 5 Mental Health and Behavioral Sciences, James A. Haley Veterans' Hospital, Tampa, USA; 6 Department of Psychiatry, University of Florida College of Medicine, Gainesville, USA

**Keywords:** buprenorphine, substance use screening, medication-assisted treatment, drug-drug interactions, herbal medicine use, alternative medicine, opioid use disorder (oud), cyp 3a4, herba epimedii

## Abstract

Herba Epimedii, commonly known as yin yang huo, inyokaku, and horny goat weed, is a traditional Chinese herbal medicine utilized for treating osteoporosis and enhancing libido. Studies conducted in vitro have demonstrated that Herba Epimedii interacts with the enzyme cytochrome P450 3A4 (CYP3A4). This interaction poses a potential risk for drug-drug interactions, particularly with medications metabolized by CYP3A4, such as buprenorphine. This paper presents a case of a patient experiencing exacerbated opioid cravings following the initiation of Herba Epimedii. This is the first reported case supporting this interaction, emphasizing the necessity of screening for alternative medicines in patients undergoing medication-assisted treatments for opioid use disorder.

## Introduction

The use of herbal medicines in the United States has grown in popularity, with studies indicating that between 20% and 33% of patients report using such remedies to their provider [1,2]. Herbal medicines are utilized for a wide range of maladies but most often are used for the common cold, musculoskeletal conditions, and gastrointestinal problems. Factors associated with higher use of herbal medicines include cultural factors, being uninsured, having a higher education level, and being aged between 45-64 years old [1]. Notably, up to 72% of herbal medicine users have reported taking these remedies with prescription medications, yet only half of these individuals disclose their use of herbal medicine to their healthcare providers [1]. 

Herba Epimedii, a traditional Chinese herbal medicine, is derived from the dried leaves of multiple species of Epimedii. Herba Epimedii has historically been used to improve libido and skeletal health, with recent literature highlighting its benefits in cardiovascular diseases, osteoporosis, libido, and dementia [3]. Its potential effectiveness in treating erectile dysfunction is thought to be due to icariin, an active component that inhibits human phosphodiesterase 5, the same mechanism of action observed in sildenafil [4]. Epimedium brevicornum, a specific Epimedii species, has been shown to induce cytochrome P450 3A4 (CYP3A4) through activation of the pregnane X receptor pathway [5]. However, these findings are limited to cell-based studies. In this report, we present a case involving a suspected interaction between Herba Epimedii and buprenorphine, a partial opioid agonist utilized in opioid use disorder that is also metabolized by CYP3A4. 

## Case presentation

The patient, a 62-year-old male with a history of opioid use disorder in sustained remission, presented to the outpatient clinic for management of opioid use with medication-assisted treatment in the context of increased opioid cravings. During the interview, the patient described experiencing worsening opioid cravings over the past few weeks, which would start in the middle of the night and last until his next morning dose of buprenorphine/naloxone (Suboxone®). The patient had maintained sobriety from opioids for ten years with daily buprenorphine/naloxone 8 mg/2 mg. Since the initiation of buprenorphine/naloxone, this dose has adequately managed opioid cravings. He denied any changes to the way he had been taking his buprenorphine/naloxone. He denied any recent relapses, which was confirmed with a negative urine drug screen and positive urine buprenorphine level just prior to presentation. 

This patient was also being treated for schizoaffective disorder, bipolar type, by an outside provider and was taking the following medications: quetiapine 600 mg nightly, sertraline 50 mg daily, lamotrigine 100 mg twice daily, topiramate 200 mg twice daily, and hydroxyzine 25 mg nightly as needed for insomnia. There were no recent changes in these psychotropic medications. On review of alternative medication use, the patient noted starting a new supplement, Herba Epimedii, earlier that month to increase his libido. The patient started Herba Epimedii as he had gotten into a new relationship and saw this could be helpful after searching online for libido remedies. The diagnostic work-up, which included a complete blood count and a comprehensive metabolic panel, was otherwise unremarkable.

Following the outpatient visit, the Suboxone® dose was thus titrated from 8 mg/2 mg to 10 mg/2.5 mg daily. The patient reported an improvement in opioid cravings within days to weeks of starting the increased dosage. The patient's enhanced control over his opioid cravings was sustained, as evidenced during the six-month follow-up visit.

## Discussion

Buprenorphine is a medication used for opioid use disorder treatment, opioid withdrawal management, and pain management. It functions as a partial mu-opioid agonist and binds with high affinity to the mu-opioid receptor, resulting in a reduction of opioid cravings. Buprenorphine is available as an office-based opioid treatment, with patients going to the clinic for weekly or monthly refills [6]. To be effective, buprenorphine should be titrated to a reduction of cravings [7]. Buprenorphine is metabolized by CYP3A4 in the liver, leading to caution in use with patients who have liver dysfunction or who are on medications including macrolides, HIV protease inhibitors, and azoles [6].

Individuals with opioid use disorders are susceptible to trialing alternative therapies and herbal medicines. Rates of using herbal medicines are higher in patients taking buprenorphine for opioid use disorder and chronic pain when compared to patients who do not meet the criteria for chronic pain [8]. Additionally, long-standing use of opioids has been associated with hypogonadism, leaving this population more vulnerable to turning to alternative medicines like Herba Epimedii to manage decreased libido and erectile dysfunction [9].

In this case, Herba Epimedii's proposed effect on CYP3A4 is supported by the temporal nature of increased opioid cravings and decreased buprenorphine effectiveness after starting Herba Epimedii. It is less likely that this was a spontaneous development of tolerance to the medication, as the patient was on a consistent dose of Suboxone® for ten years, with a consistent lack of cravings and dependence. Limitations of this finding include the reliance on the patient's self-reported opioid cravings and the absence of objective signs of opioid withdrawal accompanying these cravings. The patient was also on other medications metabolized by CYP3A4, including quetiapine, hydroxyzine, and sertraline. However, no changes to these medications had been made for the three months prior to the initiation of Herba Epimedii and the onset of opioid cravings. Lastly, the inability to determine what combination of species of Herba Epimedii was included in the supplement the patient purchased constrains our ability to ascertain whether it contained Epimedium brevicornum or a different species not previously studied.

Other supplements with a similar interaction are listed in Table 1. These include Echinacea (Echinacea purpurea), which is typically used in upper respiratory tract infections; Saint John's wort (Hypericum perforatum), which is used for depression; and Ginseng (Panax ginseng), which is often used for mental performance and diabetes [10,11]. We therefore recommend routine screening for herbal supplements in patients with opioid use disorder, particularly those on buprenorphine and other CYP3A4-metabolized treatments, to prevent similar drug-drug interactions.

**Table 1:** Buprenorphine use and additional potential supplement interactions CYP3A4 - cytochrome P450 3A4 Source: [10]

CYP3A4 modulators	Dietary supplements	Potential impact on buprenorphine dosing
CYP3A4 inhibitors	Grapefruit (Citrus paradisi); Goldenseal (Hydrastis canadensis); Peppermint (Mentha piperita); Cranberry (Vaccinium macrocarpon)	Consider decreased buprenorphine dosing (while ensuring opioid cravings are managed).
CYP3A4 inducers	Echinacea (Echinacea purpurea); Saint John's wort (Hypericum perforatum); Ginseng (Panax ginseng)	Consider increased buprenorphine dosing to manage opioid cravings.

## Conclusions

This case report reinforces the importance of screening for alternative medications and supplements, which can affect the metabolism of medication-assisted treatments (MAT). The unregulated status of herbal supplements, coupled with low supplement disclosure rates, poses risks of interactions with prescribed medications, unanticipated and adverse side effects, and complications in managing comprehensive treatment plans. Considering the heightened relapse risk when buprenorphine is inadequately adjusted to mitigate cravings and the associated overdose risk with relapse, it is critical to consistently monitor for any new alternative medications or supplements, especially when there is a noticeable change in the effectiveness of the medication.
